# The Modern Medical Bureaucrat

**Published:** 1914-04-18

**Authors:** 


### THE MODERN MEDICAL BUREAUCRAT.

The general aspects of our Fabian friends' estimate of the shortage of hospitals we have already noticed. Inviting them in passing to bear in mind that the long waiting lists of patients they speak of are nearly all people waiting for surgical operations, and to talk, therefore, in future a little more kindly of surgeons, we now turn to something in connection with their suggested, or rather adumbrated, remedy for the shortage aforesaid. It is indicated in the observation that under the Public Health Acts there is technically power for a local sanitary authority to establish and maintain out of the rates hospitals for any diseases and any patients whatsoever, with or without charge. In short, they look to "the stream of municipal progress" which "flows on, almost unnoticed, through the shifting sands of party politics." Now that tendency may, or may not, be a very good thing in general principle, but when it comes to hospitals there happens to be an important accident of the particular case.

Let us see how municipal activity in matters medical has rightly increased and multiplied of late. First came school, then tuberculosis, medical officers; now oculists and dentists are being appointed. Who supervises these gentlemen—who, by the credulity of laymen regarding a prosperous and middle-aged medical man, is supposed to know all about their duties? The answer is, of course, the medical officer of health; who yet has done none of the investigation which has furnished the fresh knowledge in the light of which their services are required. And if municipal general hospitals were established, and the question put to whom their staffs should be considered assistants, we make small doubt but that Fabians would answer the medical officer of health likewise. Their school of thought considers the sanitary officer the single type of medical man who is not "a parasite on disease"; and being of a general anti-vivisectionist complexion, it has remained uninterested by the results of medical research and quite unaware of the difficulties encountered, and the huge efforts and devotion expended, in this pursuit. They do not know that the only manifestation of the fact that most doctors are "financially interested" in disease is a lively rivalry as regards therapeutics. They do not know that the way to find out about disease is to stick to it as closely as a parasite does. In fact, they have hold of the wrong end of the medical stick, and (to revert to our particular point) would clearly be surprised to learn that as a general thing the abler members of the profession become hospital clinicians or pathologists, while the Services, sanitary, colonial and combatant, are recruited from the rest.

Already some medical officers of health have charge of more than they can properly understand, and so far from their burdens being increased, there should in a year or two, if things are to fall out for the best, be begun a process of "hiving off." Naturally, one can hardly expect the initiation of this process to come from medical officers of health themselves. In fact, there is a story of one of them, at the time of the revolt against the Insurance Act, coming up to London full of a project of a municipal medical agency for the supply and direction of doctors to take on the general medical practice of his town. He had even thought of a municipal medical school, for which, in the words of Sir Wm. Savory's dry reply to an ambitious provincial surgeon, he was no doubt ready to procure "a skeleton and a microscope." In so far as municipal hospitals would mean a fuller throwing open of the medical career to talent, they would on the professional (and therefore also on the social) side mark a great advance. But to institute something like a bureaucracy, which is inevitable where the highest position is only open to administrators; to substitute the spirit of the office for that of science; to wait on instructions from Whitehall instead of fearlessly taking responsibility—these things would mean a sorry change for the worse. No one denies that doctors are the servants of the community. But the best of them know best how that community should, and should not, be medically served.

